# Comparative evaluation of single‐artery cannulation with passive venous drainage versus traditional dual‐cannula ex vivo lung perfusion in a rat model

**DOI:** 10.1002/ame2.70122

**Published:** 2026-01-18

**Authors:** Ming Ni, Fei Xue, Xuanpeng Wu, Chenxi Li, Shuhao Liang, Tianhao Chen, Leyu Hong, Chao Luo, Tong Liu, Jingyao Zhang, Chang Liu, Qifei Wu

**Affiliations:** ^1^ Department of Thoracic Surgery The First Affiliated Hospital of Xi'an Jiaotong University Xi'an China; ^2^ Key Laboratory of Surgical Critical Care and Life Support Xi'an Jiaotong University, Ministry of Education Xi'an China

**Keywords:** ex vivo lung perfusion (EVLP), lung preservation, lung transplantation, passive venous drainage

## Abstract

**Background:**

Ex vivo lung perfusion (EVLP) has emerged as a critical technique for lung preservation and evaluation prior to transplantation. While conventional rat EVLP systems utilize closed‐loop dual cannulation of pulmonary artery (PA) and vein, the effect of the simplified model using single PA cannulation with passive venous drainage is unknown.

**Methods:**

We developed two EVLP models in rats: a semi‐closed circuit with PA‐only cannulation and left atrial incision for passive venous drainage (SC‐EVLP), and a closed circuit employing both arterial and venous cannulation (C‐EVLP). Donor lungs were perfused for a defined duration and subsequently orthotopically transplanted. We evaluated pulmonary function parameters, histopathological injury scores, inflammatory cytokine levels, and apoptotic marker expression at the end of perfusion and posttransplantation.

**Results:**

Compared to the conventional EVLP, the SC‐EVLP group exhibited significantly lower PA pressure and improved dynamic lung compliance throughout perfusion. Although the levels of tumor necrosis factor‐α in the perfusate were higher in the SC‐EVLP group, other cytokine levels in the perfusate and bronchoalveolar lavage fluid exhibited no significant differences. Pulmonary edema was reduced in the SC‐EVLP group, as indicated by a lower lung wet‐to‐dry ratio. After transplantation, lungs from the SC‐EVLP group exhibited lower histological injury scores, reduced apoptosis, and decreased serum cytokine levels, suggesting attenuated inflammation and tissue damage.

**Conclusions:**

In a rat model, single PA cannulation with passive venous drainage reduced pulmonary edema during EVLP and reduced lung injury and systemic inflammation after transplantation.

## Graphical abstract

This study compared a simplified rat ex vivo lung perfusion model with single‐artery inflow and passive vein drainage to the traditional dual‐catheter closed‐loop system.

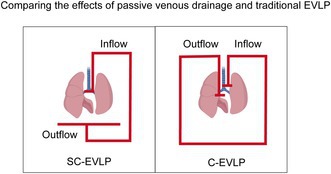

## 1 INTRODUCTION

Ex vivo lung perfusion (EVLP) has emerged as a valuable platform for preserving, reconditioning, and evaluating donor lungs prior to transplantation.^1^, ^2^ By maintaining isolated lungs in a physiologically supportive environment outside the body, EVLP allows for both functional assessment and therapeutic intervention, thereby expanding the pool of transplantable organs.^3^

**FIGURE 1:**
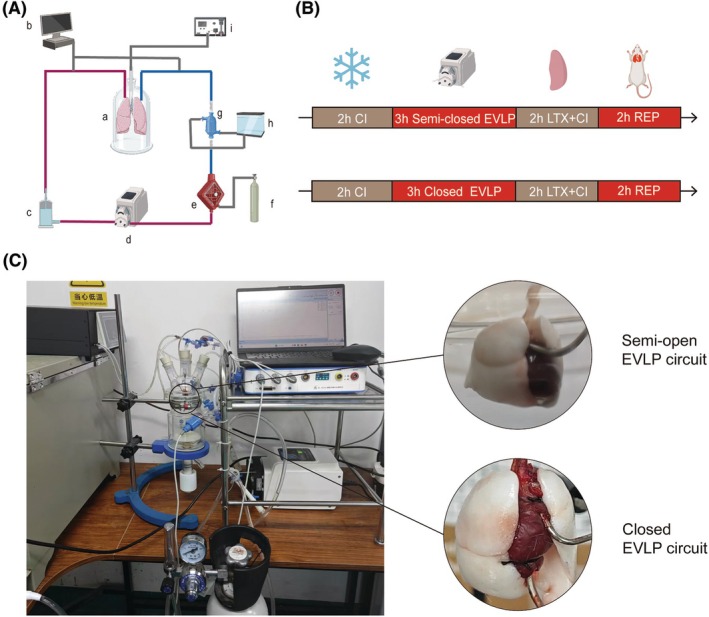
Introduction of EVLP (ex vivo lung perfusion) system and experimental design. (A) The schematic diagram of EVLP model establishment. (B) Study design. (C) Images of the semi‐closed EVLP (SC‐EVLP) setup and the closed EVLP (C‐EVLP) setup. CI, cold ischemia; LTx, lung transplantation; REP, reperfusion. a, donor lung; b, pressure monitor; c, reservoir; d, pump; e, gas exchange membrane; f, gas mixture tank; g, heat exchanger; h, water tank; i, ventilator.

Traditional EVLP systems rely on a closed perfusion circuit, in which both the pulmonary artery (PA) and pulmonary vein (PV) are cannulated to establish a recirculating loop (Figure 1A).^4^, ^5^, ^6^ Although this configuration effectively maintains perfusate flow and enables controlled gas exchange, it also presents physiological and technical challenges.^7^, ^8^ In particular, during our EVLP experiments in rats, we observed that the closed‐loop design transmits negative pressure from the pump to the pulmonary venous system, especially during prolonged perfusion. This retrograde suction effect can lead to venous congestion and pulmonary edema, compromising lung integrity and gas exchange capacity.

To address these limitations, we developed a semi‐open EVLP system in which only the PA is cannulated, and the venous outflow is left free to drain by gravity. This simplified design reduces circuit complexity and avoids the production of negative pressure in the PVs, thereby minimizing venous backpressure and potentially improving the feasibility of long‐term perfusion. However, the exposure of perfusate to ambient air and nonbiological surfaces in a semi‐open configuration may enhance inflammatory activation, a factor that remains poorly characterized.^9^, ^10^

This study aims to evaluate the lung mass, inflammatory response, and posttransplantation function after perfusion using semi‐open or closed EVLP circuits in rat models in an effort to determine the trade‐offs between ease of operation and physiological protection for each method (Figure 1B).

## 2 MATERIALS AND METHODS

### 2.1 Animals

For the experiment, male Sprague–Dawley special pathogen‐free rats, weighing 300–320 g, were purchased from the Experimental Animal Center of Xi'an Jiaotong University. The rats were housed in groups and allowed a period to acclimatize to the laboratory environment before the study began. All animal procedures were performed in accordance with the National Institutes of Health (NIH) Guide for the Care and Use of Laboratory Animals or its equivalent. This study was approved by the Ethics Committee of the First Affiliated Hospital of Xi'an Jiaotong University.

### 2.2 Donor lung procurement

Male rats were anesthetized with an intraperitoneal injection of pentobarbital sodium (50 mg/kg) and underwent tracheotomy followed by mechanical ventilation (Alcott Biotech, ALC‐V8S‐631, Shanghai, China). Ventilation parameters were set to a tidal volume (Vt) of 7 mL/kg, a respiratory rate (RR) of 70 breaths/min, and an inspiratory‐to‐expiratory ratio of 45:55. After laparotomy and sternotomy, 300 IU of heparin (Xinbai, Nanjing, China) was administered intravenously via the inferior vena cava. The PA was cannulated by making an incision in the right ventricle. The semi‐closed group (SC‐EVLP) had the left atrial appendage incised, but no PV catheterization was performed. In the closed‐circuit group (C‐EVLP), the conventional EVLP procedure was used. The left atrium was cannulated by retrograde insertion through the mitral valve via the left ventricle. After catheterization, the rats were killed by administering an overdose of pentobarbital sodium and then exsanguinated through the inferior vena cava (Figure 1C).^11^ Each group consisted of seven rats.

Next, 20 mL of cold (4°C) Perfadex solution was infused into the PA to flush the donor lung. During this process, ventilation was maintained at an RR of 30 breaths/min with a Vt of 7 mL/kg. Next, the lungs were inflated to a sustained airway pressure of 15 cmH_2_O, and the trachea was clamped. The heart–lung block was then excised, weighed, and immediately immersed in Perfadex solution at 4°C for 2 h.^12^

### 2.3 EVLP and physiological variables

The EVLP system was constructed using commercially available small‐animal experimental instruments. Before perfusion, 30 mL of perfusate prepared according to the formula of Wei et al.^13^ was preloaded into the circuit, and 40 mg of methylprednisolone, 40 mg of cephalosporin, and 300 units of heparin were added. A peristaltic pump maintained the perfusion flow at 20% of the calculated cardiac output (75 mL/min per 250 g body weight) to minimize pulmonary edema, ensuring that the mean PA pressure remained <15 mmHg.^14^ An oxygenator deoxygenated the perfusate using a gas mixture of 8% CO_2_, 6% O_2_, and 86% N_2_, allowing lung function assessment via effluent oxygen content. The perfusate temperature was gradually increased to 37°C over 30 min before mechanical ventilation. Ventilation settings included a tidal volume of 7 mL/kg, an inspiratory‐to‐expiratory ratio of 45:55, an RR of 30 breaths/min, and a positive end‐expiratory pressure (PEEP) of 3 cmH_2_O.^15^, ^16^, ^17^

PA and PV and airway pressures were continuously monitored (Techman, BL420N, Chengdu, China). The PV pressure of the SC‐EVLP group was denoted as 0 mmHg. Lung compliance was calculated as tidal volume divided by the difference between inspiratory plateau pressure and PEEP. Every hour, 1 mL of perfusate was sampled for blood gas analysis (P/F ratio). To standardize oxygenation conditions prior to sampling, the oxygen concentration was increased to 100% 5 min before each collection. After perfusion, different lobes of the right lung were allocated for specific analyses: the upper lobe was used to collect bronchoalveolar lavage fluid (BALF),^12^ the middle lobe was used for calculating the wet‐to‐dry weight ratio (W/D ratio), and the lower lobe was reserved for histological examination. The left lung was preserved for transplantation.

### 2.4 Left lung transplantation

After EVLP preservation, the donor left lung was transplanted into a recipient male Sprague–Dawley rat using the nonsuturing external cuff technique described by Mizobuchi et al.^18^ Briefly, the PV, PA, and left main bronchus were passed through polytetrafluoroethylene cuffs, which were then everted and secured with 8–0 Prolene sutures. The PA and PV cuffs were made using 18G catheters, whereas the tracheal cuff was made using a 16G catheter.^19^, ^20^, ^21^

The recipient rat was anesthetized using the same protocol as described earlier. The trachea was exposed and incised to allow mechanical ventilation (FiO_2_, 0.21; RR, 70 breaths/min; tidal volume, 7 mL/kg; and PEEP, 3 cmH_2_O). A left posterolateral thoracotomy was performed to access the hilum. The PA, PV, and bronchus were dissected and clamped. Small incisions were made in each structure, and vascular and bronchial cuffs were inserted and secured with 8–0 Prolene sutures. The clamp was then released to restore reperfusion and ventilation. The lung gradually turned red and expanded and contracted in rhythm with the ventilator. The chest incision was then closed (Video S1). After 2 h of reperfusion, the rats were killed with an overdose of pentobarbital sodium, and the transplanted left lungs and blood serum were collected. There were five rats in each group that had undergone the lung transplantation procedure.

### 2.5 Inflammatory marker analysis

Cytokines (tumor necrosis factor [TNF‐α], interleukin [IL]‐1β and IL‐6) in perfusate, BALF, and serum were measured using enzyme‐linked immunosorbent assay (ELISA) kits (Yuanju Bio, Shanghai, China) after operation.

### 2.6 Bronchoalveolar lavage fluid collection

After the right upper lobe was isolated, 1 mL of normal saline was administered through the bronchus using a blunt‐tipped syringe. The lung was gently compressed to recover the fluid, which was then readministered. This lavage procedure was repeated thrice, yielding ~0.8 mL of BALF.^12^

### 2.7 W/D ratio

The wet weight was measured within 5 min after harvesting, and the dry weight was measured after desiccation for 24 h at 60°C.^22^ The ratio was calculated by dividing the wet weight by the dry weight.

### 2.8 Histological and apoptosis assessment

Lung tissues were fixed in 10% buffered formalin, embedded in paraffin, and sectioned onto slides for hematoxylin and eosin (H&E) staining. Two pathologists independently and blindly evaluated lung injury using a standardized histological scoring system, with the final score representing the average of both assessments. Injury severity was graded on a scale from 0 to 4 for each of the following features: intra‐alveolar edema, intra‐alveolar hemorrhage, capillary congestion, and leukocyte infiltration. The grading scale was defined as follows: 0: 0% involvement, 1: 1%–25%, 2: 26%–50%, 3: 51%–75%, and 4: 76%–100%. The total lung injury score was calculated as the sum of the individual scores for each pathological feature.^23^

Terminal deoxynucleotidyl transferase dUTP nick‐end labeling (TUNEL) staining was utilized to assess apoptosis in lung tissue post‐EVLP, using the TUNEL apoptosis assay kit (Roche, Basel, Switzerland). TUNEL‐positive green cells and blue‐stained nuclei were counted using Image J software (NIH, USA). The results were expressed as the ratio of TUNEL‐positive cells to the total number of cells.^13^

### 2.9 Statistical analysis

Statistical analyses were performed using SPSS Statistics (version 25.0, Chicago, IL, USA). Data are shown as mean ± standard deviation. The differences between groups were compared using unpaired Student's *t*‐test or the Kruskall–Wallis test. For PA pressure and dynamic compliance and P/F ratio, we additionally performed a repeated‐measures analysis of variance (ANOVA) with assumed sphericity. A value of *p* < 0.05 was identified as statistically significant.

## 3 RESULTS

### 3.1 Lung physiology during EVLP

**FIGURE 2:**
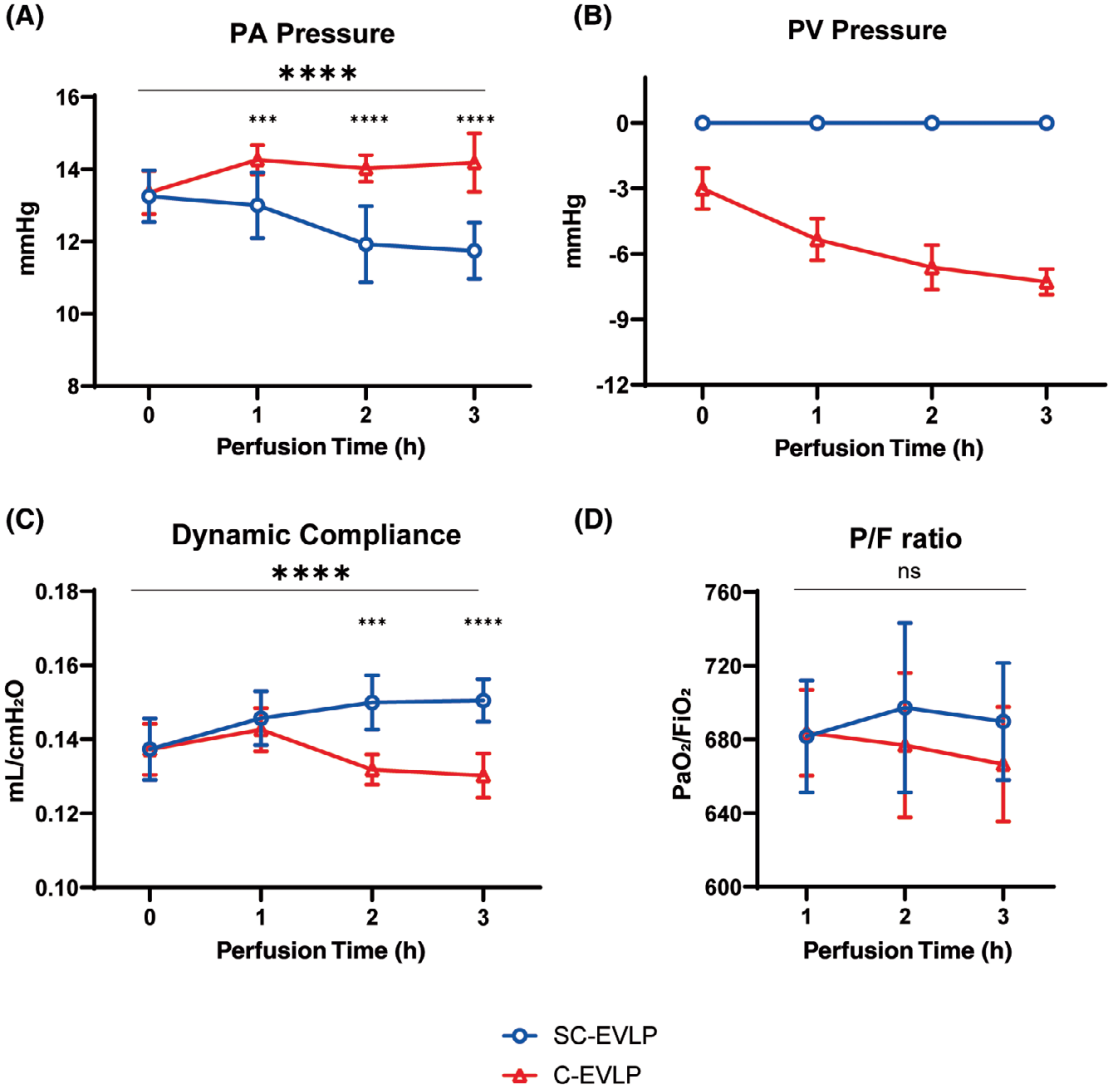
Time‐dependent physiologic changes during EVLP (ex vivo lung perfusion). (A) Hourly PA (pulmonary artery) pressure during EVLP in both the SC‐EVLP (semi‐closed EVLP) and C‐EVLP (closed‐circuit EVLP) groups. (B) PV (pulmonary vein) pressure. (C) Dynamic compliance. (D) P/F ratio. Data are presented as mean ± SD (standard deviation). Pairwise comparisons at each time point were performed using the *t*‐test or rank‐sum test, as appropriate. Overall differences between groups over time were analyzed using repeated‐measures ANOVA (analysis of variance). *n* = 7. ***p < 0.001 and ****p < 0.0001. FiO_2_, fraction of inspired oxygen; PA, pulmonary artery; P/F, partial pressure of arterial oxygenation to fraction of inspired oxygen ratio; PaO_2_, partial pressure of arterial oxygen; PV, pulmonary vain.

Throughout the EVLP procedure, the SC‐EVLP group demonstrated a progressive decrease in PA pressure. A significant difference compared to the C‐EVLP group emerged as early as the first hour (13.00 ± 0.91 vs. 14.26 ± 0.41 mmHg, *p* < 0.001) and was sustained throughout perfusion (Figure 2A). In contrast, PV negative pressure in the C‐EVLP group gradually increased over time (Figure 2B). In terms of lung compliance, the SC‐EVLP group exhibited a gradual increase over time, whereas the C‐EVLP group exhibited a decrease beginning at the 2‐h mark. A significant difference in compliance was first observed at 2 h (0.1500 ± 0.0073 vs. 0.1318 ± 0.0041 mL/cmH_2_O, *p* < 0.001) and remained significant through the end of the procedure (Figure 2C). Repeated‐measures ANOVA confirmed significant overall differences in both PA pressure and dynamic compliance between the groups. However, there was no significant difference in the P/F ratio (Figure 2D).

### 3.2 Histological assessment and apoptosis after EVLP

**FIGURE 3:**
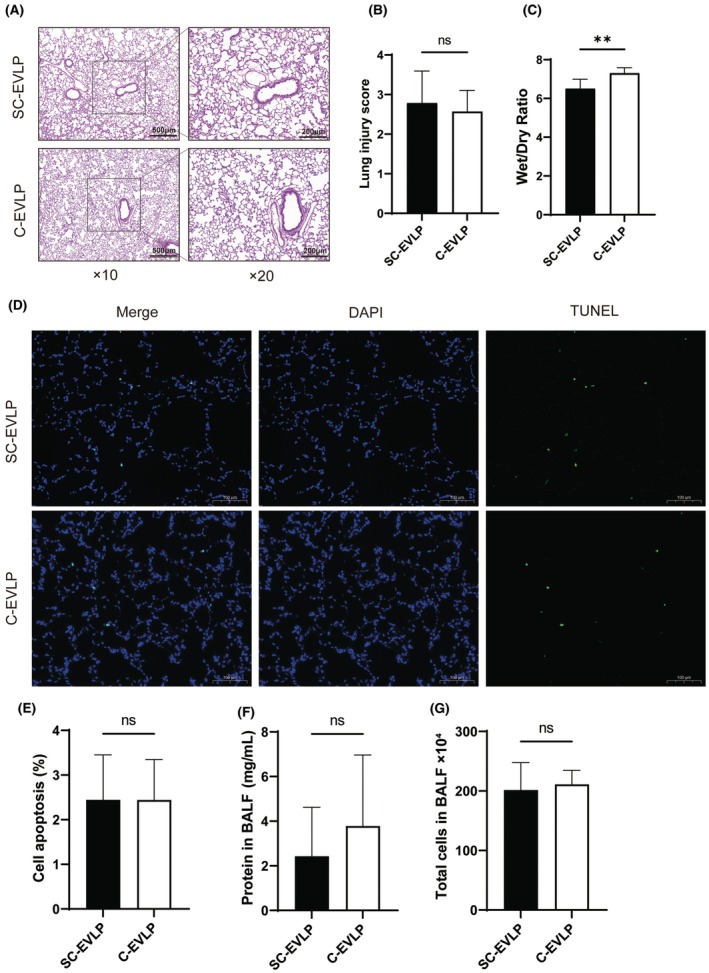
Comparison of lung injury markers after EVLP (ex vivo lung perfusion) between SC‐EVLP (semi‐closed EVLP) and C‐EVLP (closed‐circuit EVLP) groups. (A and B) Representative H&E (hematoxylin and eosin)–stained lung sections and corresponding lung injury scores. (C) Dry‐to‐wet weight ratio of the right lung, indicating tissue edema. (D and E) TUNEL (terminal deoxynucleotidyl transferase dUTP nick‐end labeling) staining of lung sections with quantification of apoptotic (TUNEL‐positive) cells. (F) Total protein concentration in BALF. (G) Total cell count in BALF. *n* = 7. **p < 0.01. BALF, bronchoalveolar lavage fluid.

H&E staining of right lung tissue after EVLP exhibited no significant differences between the SC‐EVLP and C‐EVLP groups (2.786 ± 0.809 vs. 2.571 ± 0.535, *p* = 0.57) in terms of intra‐alveolar edema, hemorrhage, capillary congestion, or leukocyte infiltration (Figure 3A,B). However, the SC‐EVLP group exhibited a lower W/D ratio (6.51 ± 0.48 vs. 7.30 ± 0.28, *p* < 0.01), indicating less pulmonary edema—potentially due to the absence of negative pressure in the PVs (Figure 3C). The proportion of apoptotic cells was also similar between the two groups (2.446% ± 1.007% vs. 2.441% ± 0.908%, *p* = 0.99; Figure 3D,E). Although the SC‐EVLP group also exhibited lower protein levels and total cell counts in BALF, these differences were not statistically significant (Figure 3F,G).

### 3.3 Inflammatory response after EVLP

**FIGURE 4:**
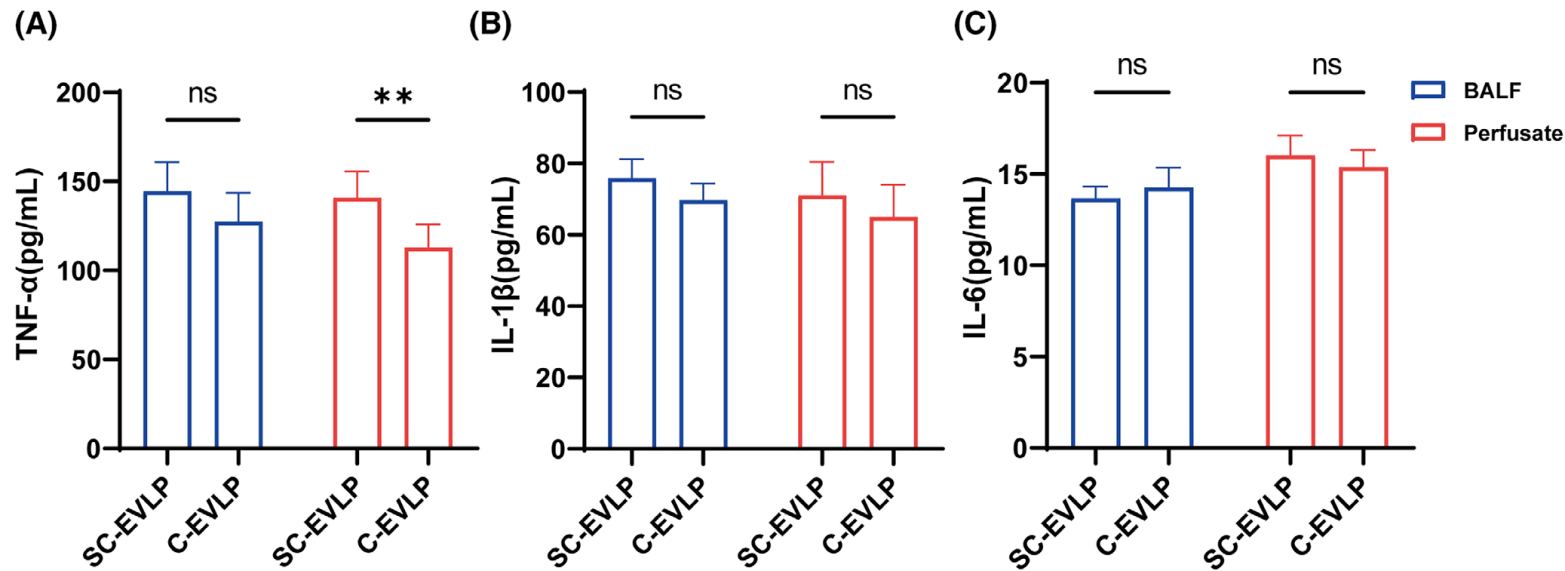
Cytokine concentrations in BALF (bronchoalveolar lavage fluid) and perfusate after EVLP (ex vivo lung perfusion). (A) TNF‐α. (B) IL‐1β. (C) IL‐6. *n* = 7. **p < 0.01. IL, interleukin; TNF, tumor necrosis factor.

Cytokine levels (TNF‐α, IL‐1β, and IL‐6) were measured in both the perfusate and BALF from the right upper lobe after EVLP. Among these, only TNF‐α levels in the perfusate were significantly higher in the SC‐EVLP group compared to the C‐EVLP group (140.68 ± 13.74 vs. 112.82 ± 12.20 pg/mL, *p* < 0.05; Figure 4A). Although TNF‐α and IL‐1β levels in BALF, as well as IL‐1β and IL‐6 levels in the perfusate, exhibited an upward trend in the SC‐EVLP group, these differences did not reach statistical significance (Figure 4A–C).

### 3.4 Posttransplantation histology and arterial blood gas

**FIGURE 5:**
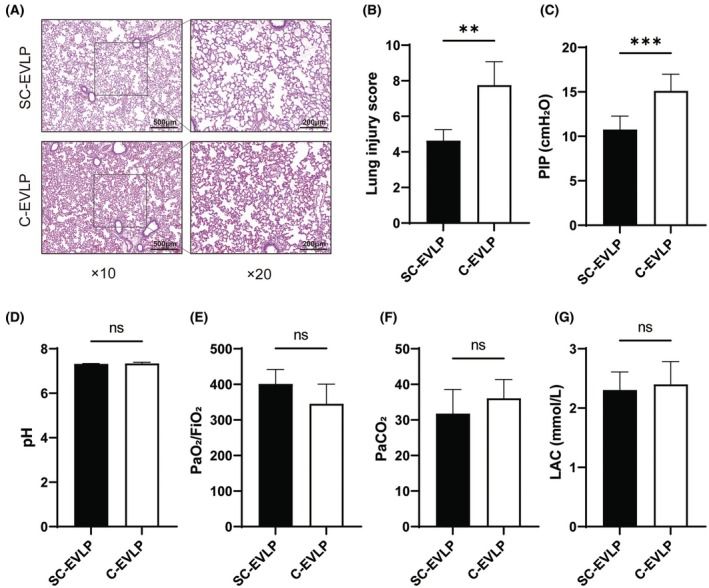
Histological and functional assessment of the left lung after transplantation. (A and B) Representative H&E (hematoxylin and eosin)–stained lung sections and corresponding lung injury scores. (C) Comparison of PIP. (D–G) Blood gas analysis 2 h after transplantation, including (D) pH, (E) P/F ratio, (F) PaCO_2_, and (G) LAC, reflecting recipient lung function. *n* = 5. **p < 0.01 and ***p < 0.001. LAC, lactate; PaCO_2_, partial pressure of carbon dioxide; P/F, arterial oxygen tension to inspired oxygen fraction ratio; PIP, peak inspiratory pressure.

Two hours after reperfusion, H&E staining of the transplanted lungs showed that the SC‐EVLP group had reduced intra‐alveolar edema, hemorrhage, capillary congestion, and leukocyte infiltration compared to the C‐EVLP group. The overall lung injury score was significantly lower in the SC‐EVLP group (4.63 ± 0.63 vs. 7.75 ± 1.32, *p* < 0.05; Figure 5A,B). Additionally, peak inspiratory pressure was lower, indicating reduced airway resistance (10.76 ± 1.51 vs. 15.11 ± 1.88, *p* < 0.001; Figure 5C). However, arterial blood gas analysis revealed no significant differences in pH, P/F ratio, PaCO_2_, or lactate levels between the two groups, possibly due to compensatory function of the native right lung in recipient rats (Figure 5D–G).

### 3.5 Posttransplantation apoptosis and inflammatory response

**FIGURE 6:**
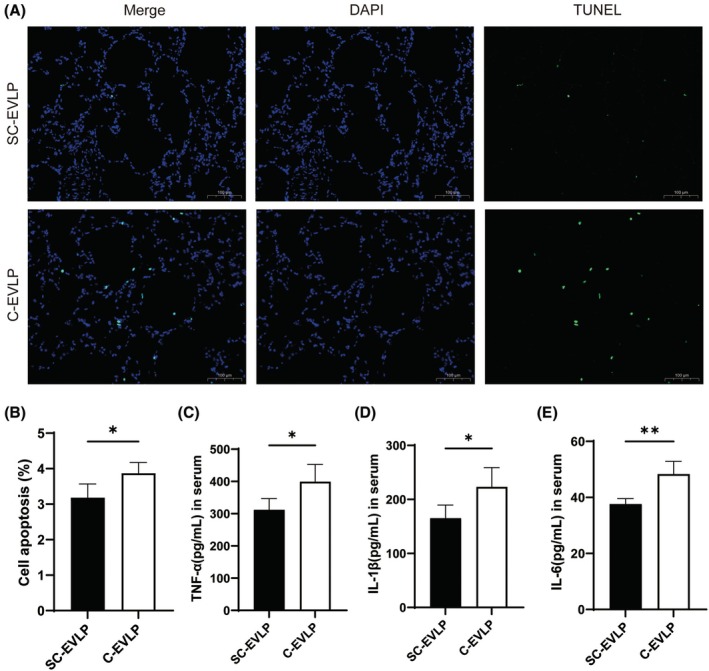
Apoptosis in the left lung and serum cytokine levels after transplantation. (A and B) TUNEL (terminal deoxynucleotidyl transferase dUTP nick‐end labeling) staining of lung sections and quantification of apoptotic cells. (C–E) Serum levels of cytokines in recipient rats, including (C) TNF‐α (tumor necrosis factor‐α), (D) IL‐1β (interleukin‐1β), and (E) IL‐6. *n* = 5. *p < 0.05 and **p < 0.01.

Two hours after transplantation, the SC‐EVLP group exhibited a significantly lower proportion of TUNEL‐positive cells (3.18% ± 0.39% vs. 3.87% ± 0.30%, *p* < 0.05; Figure 6A,B). Serum levels of TNF‐α (312.0 ± 34.98 vs. 398.8 ± 53.89 pg/mL, *p* < 0.05), IL‐1β (165.18 ± 24.33 vs. 223.12 ± 35.69 pg/mL, *p* < 0.05), and IL‐6 (37.65 ± 1.95 vs. 48.31 ± 4.56 pg/mL, *p* < 0.05) were also lower in this group, suggesting a less pronounced systemic inflammatory response (Figure 6C–E).

## 4 DISCUSSION

Since its first clinical application by Steen et al.^24^ in 2007, EVLP has gained considerable attention and has become increasingly adopted in transplantation practice.^25^ Previous studies have shown that EVLP can significantly reduce donor lung injury and make marginal lungs suitable for transplantation, with posttransplant survival rates comparable to those using standard donor lungs.^26^, ^27^ However, some reports have indicated a higher incidence of postoperative complications in recipients of EVLP‐treated lungs.^28^, ^29^, ^30^ As a result, efforts to optimize EVLP protocols to improve transplant outcomes remain a central focus of ongoing research.

Recent studies have explored various strategies to enhance the protective effects of EVLP. For instance, Gloria et al.^23^ investigated the role of mild hypothermia in preserving lung function during EVLP.Other studies have demonstrated that modified perfusion solutions may improve donor lung quality.^31^, ^32^, ^33^ Whether to keep the left atrial drainage open has been examined in large‐animal EVLP studies, but results remain inconsistent. Linacre et al. reported that a closed circuit with left atrial pressure maintained at 5–7 mmHg reduced pulmonary edema and inflammatory markers,^34^ whereas Nilsson et al. found that closed circulation worsened complications.^35^ Overall, stable pulmonary venous or left atrial pressure appears critical for preserving lung function. In rat EVLP models, however, maintaining stable positive venous pressure is more challenging, likely due to smaller pulmonary blood flow and stronger suction effects from the peristaltic pump. Our findings suggest that in experimental settings where venous pressure is unstable or negative suction occurs, opening the PV for passive drainage may be a practical alternative.

Our findings suggest that single PA catheterization may offer certain advantages over the traditional dual‐catheter EVLP approach involving both the PA and PV. This simplified technique reduces procedural complexity and may shorten operation time. Although greater exposure of the perfusate to air and vessel walls in the single‐catheter setup could potentially trigger a stronger inflammatory response, our results indicate that this effect remains within acceptable limits. At the end of perfusion, cytokine levels in both the perfusate and BALF were not significantly elevated, except for TNF‐α in the perfusate, which exhibited a modest but significant increase. This limited inflammatory response may be due to the relatively low number of immune cells present in the isolated heart–lung block and their dilution in the perfusion circuit. Furthermore, regular replacement of perfusate during EVLP may help to dilute and control the accumulation of inflammatory mediators.^36^, ^37^

Pulmonary edema is a key factor limiting the prolonged use of EVLP and serves as a major indicator of lung injury.^38^ It is primarily associated with increased endothelial permeability and is reflected by elevated lung wet weight, increased protein levels in BALF, and reduced lung compliance.^39^ In our study, we employed a semi‐open extracorporeal circuit in which the inflow catheter was placed in the PA, whereas venous outflow was passive and gravity dependent, rather than part of a closed loop. This approach was associated with increased lung compliance, a higher D/W ratio at the end of perfusion, and reduced signs of pulmonary edema. These findings suggest that a semi‐open system may help mitigate edema and support longer‐duration EVLP procedures.

Finally, in recipient rats after transplantation, we observed that lungs preserved using the SC‐EVLP approach exhibited less histological damage, reduced cellular apoptosis, and a milder systemic inflammatory response compared to those in the conventional EVLP group. These findings suggest that the passive venous drainage method may offer physiological benefits for transplant recipients. However, it is important to note that these results were obtained in a rat model, and whether similar outcomes can be replicated in large‐animal models or clinical settings remains to be determined.

## 5 STUDY LIMITATIONS

This study has several limitations. First, the relatively small sample size may introduce individual variability and limit the statistical power of the findings. Second, the rat model used in this study may not fully replicate the complexity of clinical lung transplantation. Third, we focused only on short‐term outcomes after transplantation and did not assess long‐term graft function or survival. Finally, the donor lungs used were preserved for a short period under cold conditions rather than marginal lungs as used in some previous studies. This difference in donor lung quality may influence the results, and further research is needed to determine whether similar benefits of SC‐EVLP are observed with marginal donor lungs.

## 6 CONCLUSIONS

This study demonstrates that the simplified EVLP approach using single PA catheterization with passive venous drainage (SC‐EVLP) is a feasible and potentially advantageous modification of the traditional EVLP technique. Despite concerns regarding increased exposure of the perfusate to air and vessel walls, the inflammatory response remained within acceptable limits. Compared to the conventional dual‐cannulation method, SC‐EVLP more effectively preserved lung function, reduced pulmonary edema, and minimized tissue injury and systemic inflammation after transplantation.

## AUTHOR CONTRIBUTIONS

**Ming Ni:** Conceptualization; data curation; writing – original draft. **Fei Xue:** Resources; software. **Xuanpeng Wu:** Supervision; validation. **Chenxi Li:** Methodology. **Shuhao Liang:** Investigation. **Tianhao Chen:** Project administration. **Leyu Hong:** Visualization. **Chao Luo:** Formal analysis. **Tong Liu:** Methodology. **Jingyao Zhang:** Funding acquisition. **Chang Liu:** Funding acquisition. **Qifei Wu:** Funding acquisition; writing – review and editing.

## FUNDING INFORMATION

This research was funded by the Natural Science Foundation of Shaanxi Province (2024JC‐ZDXM‐49), the National Natural Science Foundation of China (No. 82472191), and the Key Science and Technology Program of Shaanxi Province (2024SF2‐GJHX‐45).

## CONFLICT OF INTEREST STATEMENT

All authors reported no conflicts of interest.

## ETHICS STATEMENT

Experiments were performed under a project license (No. XJTUAE2025‐3125) granted by the Ethics Committee of the First Affiliated Hospital of Xi'an Jiaotong University.

## Supporting information


**Video S1:** This video demonstrates how SC‐EVLP and C‐EVLP groups perform perfusion and the process of lung transplantation.

## Data Availability

The data that support the findings of this study are available from the corresponding author upon reasonable request.
